# A highly conserved regulatory element controls hematopoietic expression of *GATA-2 *in zebrafish

**DOI:** 10.1186/1471-213X-7-97

**Published:** 2007-08-20

**Authors:** Zhongan Yang, Hong Jiang, Fang Zhao, Deepa B Shankar, Kathleen M Sakamoto, Michael Q Zhang, Shuo Lin

**Affiliations:** 1 Department of Molecular, Cell and Developmental Biology, University of California Los Angeles, Los Angeles, California 90095-1606, USA; 2 Watson School of Biological Sciences, Cold Spring Harbor Laboratory, Cold Spring Harbor, NY 11724, USA; 3 Division of Hematology-Oncology and Pathology and Laboratory Medicine, Gwynne Hazen Cherry Memorial Laboratories, David Geffen School of Medicine at UCLA, Los Angeles, California 90095-1752, USA

## Abstract

**Background:**

GATA-2 is a transcription factor required for hematopoietic stem cell survival as well as for neuronal development in vertebrates. It has been shown that specific expression of *GATA-2 *in blood progenitor cells requires distal *cis*-acting regulatory elements. Identification and characterization of these elements should help elucidating transcription regulatory mechanisms of *GATA-2 *expression in hematopoietic lineage.

**Results:**

By pair-wise alignments of the zebrafish genomic sequences flanking *GATA-2 *to orthologous regions of fugu, mouse, rat and human genomes, we identified three highly conserved non-coding sequences in the genomic region flanking *GATA-2*, two upstream of *GATA-2 *and another downstream. Using both transposon and bacterial artificial chromosome mediated germline transgenic zebrafish analyses, one of the sequences was established as necessary and sufficient to direct hematopoietic GFP expression in a manner that recapitulates that of *GATA-2*. In addition, we demonstrated that this element has enhancer activity in mammalian myeloid leukemia cell lines, thus validating its functional conservation among vertebrate species. Further analysis of potential transcription factor binding sites suggested that integrity of the putative HOXA3 and LMO2 sites is required for regulating *GATA-2/GFP *hematopoietic expression.

**Conclusion:**

Regulation of *GATA-2 *expression in hematopoietic cells is likely conserved among vertebrate animals. The integrated approach described here, drawing on embryological, transgenesis and computational methods, should be generally applicable to analyze tissue-specific gene regulation involving distal DNA *cis*-acting elements.

## Background

The transcription factor GATA-2, which is expressed during the earliest stages of hematopoiesis, is essential for early hematopoietic development; *GATA-2*^-/- ^mice have severe anemia and are deficient in the proliferation and survival of multipotent hematopoietic progenitors [1,2]. Given the essential nature of *GATA-2*, the factors required for its hematopoietic expression are likely to play an important role in the initial stages of hematopoietic development. Although a few growth factors that affect *GATA-2 *expression are known [3,4], little is understood about the regulation of *GATA-2 *in hematopoietic tissues. Recent studies showed that *Oct-1*, *GATA*, and *Evi1 *factors and their binding sites were involved in regulating *GATA-2 *hematopoietic expression [4-6]. However, these binding sites are located in the proximal region of the promoter and are unlikely sufficient in directing hematopoietic expression of *GATA-2 *since several lines of evidence have shown that the regulatory elements required for *GATA-2 *hematopoietic expression are located many kilobase pairs (kbp) upstream of *GATA-2 *[7-9]. In fact, to fully rescue hematopoietic development in *GATA-2*^-/-^mice, constructs containing over one hundred kbp of genomic sequence are required [8].

We have previously utilized bacterial artificial chromosomes (BACs), which can accommodate inserts containing several hundred kbp of genomic DNA[2,8,10-12], to study regulation of *GATA-2 *in hematopoietic cells. Once a genomic fragment has been cloned into a BAC, it can be modified by insertion of a reporter gene[2,13-16]. Using multiple GFP reporter modified BAC clones, containing varied amounts of upstream and downstream genomic sequence, we have demarcated a distal genomic region that regulates hematopoietic *GATA-2 *expression in transgenic zebrafish [9].

In this report, we describe the identification and functional studies of two highly conserved non-coding sequence elements within this genomic region. Using *Tol2 *transposon cassettes containing these non-coding sequence elements linked to GFP, we have identified a 224 bps *cis*-acting element that is sufficient to drive reporter gene expression in a manner that recapitulates hematopoietic *GATA-2 *expression pattern in a stable transgenic zebrafish line. In addition, deleting this element from the modified BAC clone eliminates hematopoietic GFP expression. Further analysis by base change mutations coupled with transgenic analysis we demonstrated that the HOXA3 and LMO2 play critical roles in regulating *GATA-2 *hematopoietic expression.

## Results

### Comparative genomics analysis

**Figure 1:**
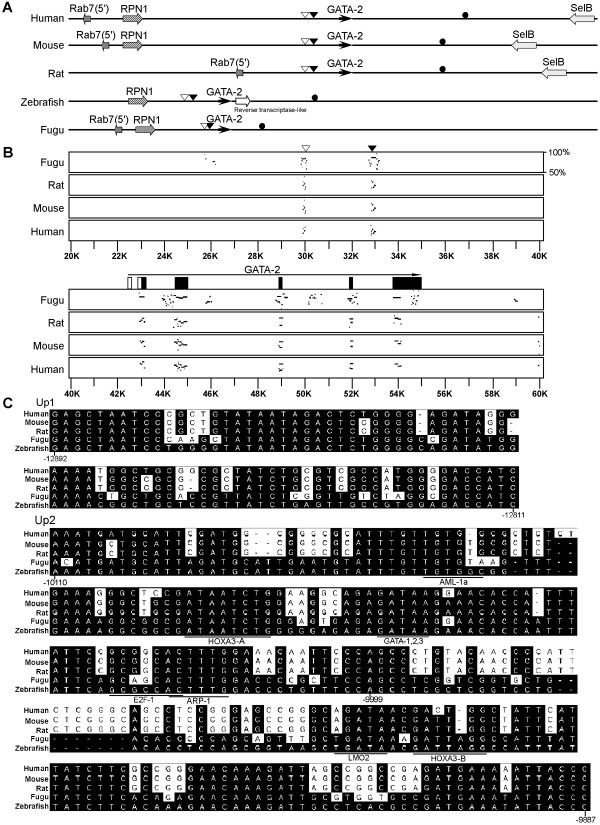
**Comparative Bioinformatics Analysis of *GATA-2 *Genomic loci**. (A) A syntenic arrangement of *GATA-2 *(solid black arrow) and *RPN1 *(diagonal striped arrow) is conserved between human, mouse, fugu and zebrafish showing that these regions are orthologous. By sequence comparison, three evolutionarily conserved elements were identified in the human, mouse, rat, fugu and zebrafish genomes, two upstream of *GATA-2*, located at 12.9 kbp (Up1, open arrowhead) and 10 kbp (Up2, solid arrowhead), and one downstream of *GATA-2 *(Down1, black dot). (B) Zebrafish *GATA-2 *locus has 6 exons (blocks). An open arrowhead indicates the location of Up1; a solid arrowhead indicates the locations of Up2 in zebrafish genomic sequence flanking *GATA-2*. Open blocks indicate the 5' UTR of zebrafish *GATA-2*; black blocks indicate zebrafish *GATA-2 *coding sequence. (C) Up1 stands for the genomic sequence lying 12892 bp to12811 bp upstream of the zebrafish *GATA-2 *start codon. The zebrafish Up1 sequence has an 81% sequence identity with the human Up1 sequence, an 80% sequence identity with both mouse and rat Up1sequences, and an 83% sequence identity with the fugu Up1 sequence. Up2 is the genomic sequence lying 10110 bp to 9887 bp upstream of the zebrafish *GATA-2 *start codon. Up2a is genomic sequence lying 10110 bp to 9999 bp upstream of *GATA-2 *start codon; Up2b is the genomic sequence lying 9998 bp to 9887 bp upstream of *GATA-2*. The zebrafish Up2 sequence has a 74%, 72%, 72% and 94% sequence identity with Up2 sequences in the human, mouse, rat and fugu genomes, respectively.

The expression patterns of *GATA-2 *in hematopoietic and neuronal tissues are conserved in vertebrates, suggesting that the arrangement and sequence of the *GATA-2 *genomic locus may be highly conserved. Comparative analyses of a158 kbp sequence spanning the zebrafish *GATA-2 *locus and approximately 400 kbp sequences of *GATA-2 *from human, mouse and rat and 190 Kb genomic sequence flanking the fugu *GATA-2 *locus has revealed a conserved syntenic relationship of *GATA-2 *with RPN1 (Figure 1A). A similar arrangement of general exon and intron structures and high sequence homology in exons has also been observed in the genomes of the five species (Figure 1B). In addition, we have identified highly conserved non-coding sequences in the genomicregion flanking *GATA-2 *(Figure 1A and 1B). We identified two conserved non-coding sequences, one at ~13 and the other ~10 kbp upstream of zebrafish *GATA-2 *start codon (Figure 1A and 1B). These sequences are located in approximately the same location in human, mouse, and rat, but in fugu are located closer to the *GATA-2 *coding sequence (4.7 Kbp and 3.8 Kbp, respectively). A conserved non-coding sequence was also found downstream of *GATA-2 *in fugu, mouse, rat, human and zebrafish genomes (Figure 1A and 1B).

The conserved sequence located between12892-12811 bp upstream of the zebrafish *GATA-2 *start codon (Up1) is 82 bp. Zebrafish Up1 sequence has an 81% sequence identity with human Up1 sequence, an 80% sequence identity with both mouse and rat Up1sequences and an 83% sequence identity with that of fugu (Figure 1C). A conserved sequence located 9887–10110 bp upstream of the *GATA-2 *start codon (Up2) is 224 bp. Zebrafish Up2 sequence has a 74%, 72%, 72% and 94% sequence identity with Up2 sequences from human, mouse, rat and fugu genomes, respectively (Figure 1C).

An alternative first exon (IS) has been found in the *GATA-2 *genomic sequences of human, rodents and chick [17-19]. In mouse genome, the conserved Up2 region (230 bp) is located at about 3 Kb 5' upstream of IS, which is approximately 4.6 Kb further upstream of the general exon IG of *GATA-2 *gene. To determine if alternative first exons also exist in zebrafish, 5' RACE was performed using mRNA from zebrafish embryos at the stages of 22~25 somites. As shown in Supplementary Figure S1 (see Additional file 1), only one PCR band was amplified, indicating that there is no alternative transcriptional product. This finding is consistent with the data from analyzing *GATA-2 *mRNA of 75% epiboly stage zebrafish embryos reported by Oren et al [4]. Although zebrafish is different, the genomic arrangement of IS among human, rodent and chick genomes are conserved.

### Functional analysis of the putative regulatory elements of GATA-2 gene in zebrafish

**Figure 2:**
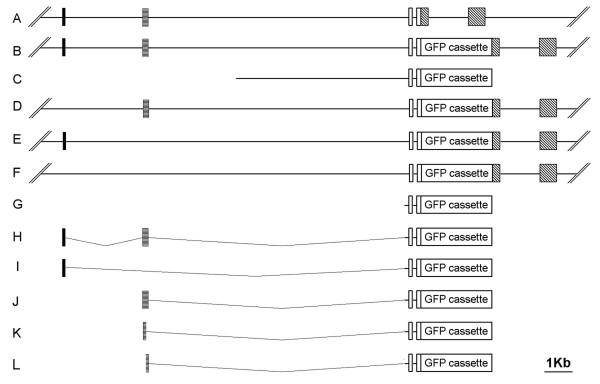
***GATA-2 *transgene constructs**. (A) A schematic diagram of the zebrafish *GATA-2 *locus shows the first three exons and approximately 14 kbp of the genomic region upstream of *GATA-2*. (B) BAC/GFP: GFP modified BAC showing reporter GFP gene inserted before *GATA-2 *translational start codon. (C) GFP reporter gene construct contains a 7.3 kbp *GATA-2 *promoter. (D) BACΔUp1/GFP is a GFP modified zebrafish *GATA-2 *BAC having a deletion of the conserved non-coding sequence Up1. (E) BACΔUp2/GFP is a GFP modified *GATA-2 *BAC having a deletion of conserved non-coding sequence Up2. (F) BACΔUp1ΔUp2/GFP is a GFP modified *GATA-2 *BAC having deletions of conserved non-coding sequences Up1 and Up2. (G) mp/GFP is a *Tol2 *plasmid vector containing GFP linked to a *GATA-2 *minimal promoter, (H) Up1Up2-mp/GFP is a *Tol2 *plasmid vector containing Up1 and Up2 linked to mp/GFP, (I) Up1-mp/GFP is a *Tol2 *plasmid vector containing Up1 linked to mp/GFP, (J) Up2-mp/GFP is a *Tol2 *plasmid vector containing linked to mp/GFP, (K) Up2a-mp/GFP is a containing Up2a linked to mp/GFP, and (L) Up2b-mp/GFP is a *Tol2 *plasmid vector containing Up2b linked to mp/GFP. In each *Tol2 *plasmid vector, GFP reporter gene constructs were inserted between *Tol2 *flanking sequences. Black block: Up1; Horizontal striped block: Up2; Open block: 5' UTR of zebrafish *GATA-2 *gene; Diagonal striped block: coding sequence of zebrafish *GATA-2 *gene.

**Figure 3:**
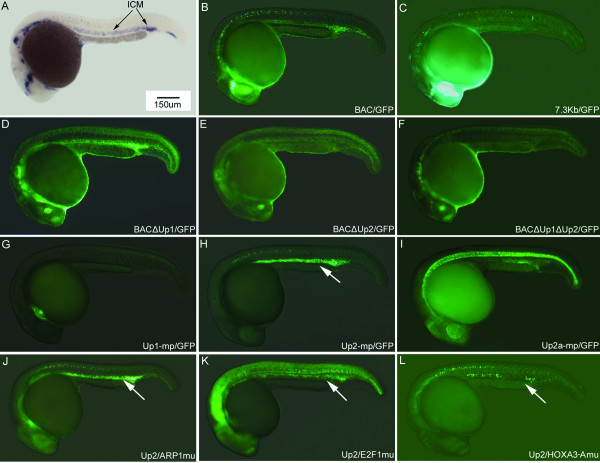
**GFP expression in *GATA-2 *transgenic zebrafish**. (A) Whole mount *in situ *hybridization shows *GATA-2 *mRNA expressed in zebrafish embryonic brain, ganglia cells, motor neurons and hematopoietic ICM region at 24 hpf stage. Arrows indicate the ICM. (B) Transgenic zebrafish carrying a modified BAC construct containing a 20 kb genomic sequence upstream of, and adjacent to the *GATA-2 *start codon show GFP expression in the ICM, brain and spinal cord at 24 hpf stage. (C) Transgenic zebrafish embryos carrying a GFP reporter gene construct linked to a 7.3 kb genomic sequence upstream of and adjacent to the *GATA-2 *start codon have strong GFP expression in neuronal cells, but not in the ICM at 24 hpf. (D) Transgenic zebrafish embryos carrying BACΔUp1/GFP have strong GFP expression in the ICM at 24 hpf stage. (E) Transgenic zebrafish embryos carrying BACΔUp2/GFP and (F) BACΔUp1ΔUp2/GFP have GFP expression in neuronal tissue but not in the ICM. (G) Transgenic zebrafish embryos carrying Up1-mp/GFP have strong GFP expression in the heart at 24 phf stage. (H) Stable transgenic zebrafish embryos containing the Up2 element linked with mp/GFP have strong GFP expression in the ICM and weak GFP expression in the spinal cord at 24 hpf stage. (I) Transgenic zebrafish embryos carrying Up2a-mp/GFP have GFP expression in ICM region at 24 hpf stage; non-specific GFP expression was observed the in notochord. (J-L) Transgenic zebrafish embryos carrying base change mutation in Up2 element derived from construct Up2-mp/GFP. (J) Mutation in the binding site of ARP1 shows no effect on the GFP hematopoietic expression. (K) Mutation in the binding site of E2F-1 results in weaker GFP expression in ICM and strong non-specific expression in muscle and notochord. (L) Mutation in HOXA3-A binding site shows significantly reduced GFP expression in ICM. Similar reduction of GFP in ICM was seen in LMO2 mutation (see Additional file 3, Supplementary Figure S2). White arrows indicate ICM expression of GFP.

As noted, during zebrafish embryogenesis, *GATA-2 *is expressed in the embryonic hematopoietic tissue Intermediate Cell Mass (ICM) and in neuronal cells (Figure 3A). ICM specific gene expression of *GATA-2 *can be recapitulated in transgenic zebrafish embryos carrying a *GFP*-modified BAC construct that includes 20 kb genomic sequence upstream of and adjacent to the *GATA-2 *start codon (Figure 2B and 3B)[9]. In contrast, GFP expression was not observed in the ICM of transgenic zebrafish embryos carrying constructs containing only 7.3 kbp of upstream sequence, although strong GFP expression was seen in neuronal cells in these embryos (Figure 2C and 3C). These results indicate that a hematopoietic regulatory element is located between 7.3 and 20 kbp upstream of the zebrafish *GATA-2 *start codon.

To determine whether conserved non-coding sequences located in this region, Up1 or Up2 are necessary, alone or in combination, to drive hematopoietic expression, we generated germline stable transgenic zebrafish carrying modified BAC constructs having deletions of Up1 or Up2 or both. In progeny of transgenic zebrafish harboring BACΔUp1/GFP, strong fluorescent expression was observed in the ICM region at 24 hpf (Figure 2D and 3D). GFP expression was stronger in embryos carrying BACΔUp1/GFP than in transgenic zebrafish embryos carrying BAC/GFP (Figure 2B and 3B). GFP expression was not observed in the ICM of embryos carrying BACΔUp2/GFP (Figure 2E and 3E) or BACΔUp1ΔUp2/GFP (Figure 2F and 3F); however, GFP expression in neuronal cells was not affected in these embryos (Figure 3E).

To determine whether these elements are sufficient, alone or in combination, to induce hematopoietic expression, GFP reporter gene constructs that contained Up1 or Up2 or both (Figure 2G–L) were used to generate transgenic zebrafish. The conserved non-coding sequences found 12.9 kb and 10 kb, respectively, upstream of the *GATA-2 *start codon were inserted into *Tol2*-transposon cassettes proximal to a *GATA-2 *minimal promoter/GFP fusion generating a series of construct combinations (Figure 2G~L). These constructs were flanked by the *Tol2 cis*-acting elements, which are required for genome integration of the construct. Each cassette was microinjected into fertilized eggs containing maternally expressed Tol2 transposase. *Tol2*-transposon mediated germline transmission of a *GATA-2 *minimal promoter/GFP reporter gene fusion (mp/GFP, Figure 2G) or of constructs with Up1, and/or Up2, inserts (Figure 2H~L) was determined by PCR. This approach, which typically produces at least 40% germline transgenic zebrafish, resulted in multiple stable transgenic zebrafish lines for each construct analyzed.

In zebrafish transgenic embryos carrying the Up1-mp/GFP construct (N = 3) (Figure 2I), no ICM-specific expression was observed; however, strong fluorescence was detected in the heart at 24 hpf (Figure 3G). In progeny of three independent transgenic zebrafish lines carrying Up2-mp/GFP, fluorescent expression was observed in blastoderm cells at 80% epiboly (data not shown). At 24 hpf, in the same transgenic lines, strong GFP expression was seen in the ICM, and weakly in the spinal cord (Figure 2J and 3H). The ICM expression pattern of Up2-mp/GFP correlates well with *GATA-2 *expression pattern determined by *in situ *hybridization (Figure 3A). Two Up2 fragments (112 bp Up2a and 112 bp Up2b, Figure 1C, Figure 2K and 2L) were also analyzed for the ability to drive hematopoietic expression in transgenic zebrafish. In four independent transgenic zebrafish lines, we found that Up2a can drive GFP expression in the ICM although less fluorescence was observed than in the Up2 transgenic and Up2a transgenic zebrafish embryos also had non-specific GFP expression in the notochord (Figure 2K and 3I). We also obtained several lines of transgenic zebrafish carrying a GFP expression vector in which both non-coding sequence elements were linked to *GATA-2 *minimal promoter (Figure 2H); GFP expression in the ICM and the heart was observed at 24 hpf (data not shown). GFP expression was undetectable in five independent transgenic zebrafish lines carrying *GATA-2 *minimal promoter construct (Figure 2G).

**Figure 4:**
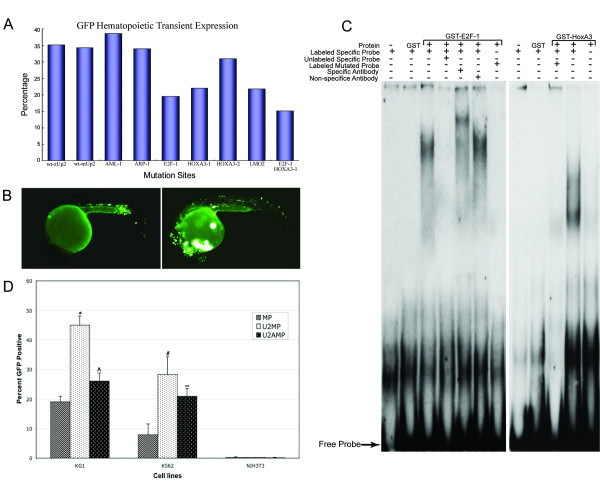
**Conservation of Up2 enhancer activity and transacting factors**. (A). Transient transgenic assays of hematopoietic GFP expression in embryos injected with wild type and mutant constructs identify HOXA3, LMO2 and E2F-1 as potential transcription factors. Also see Additional file 2, Supplementary Table S for more information about the results of hematopoietic transient expression assay. (B). Typical transient GFP expression patterns in injected embryos at 22 hpf. Left, GFP expression is detected in hematopoietic cells (arrow); Right, non-specific GFP expression was observed in muscle, heart and neuron tissue but not in hematopoietic cells (C). EMSA shows transcription factor E2F-1 (left) and HOXA3 (right) specifically bind to the probe corresponding to Up2 sequence, respectively. Probe containing HOXA3-A binding site was analyzed in EMSA. (D). Human myeloid leukemia cell lines, KG1 and K562, and mouse fibroblast cell line, NIH3T3 were transiently transfected with the mp/GFP, Up2-mp/GFP and Up2a-mp/GFP constructs. GFP expression was stronger in myeloid cells than in the fibroblast cell line. In the myeloid cell lines, enhancer activity of Up2 and Up2a was found to be statistically significant (*p < 0.002, ^p < 0.05, # p < 0.035, ** p < 0.032). The enhancer activity of Up2a/mp was less than that of Up2/mp. This figure represents the average of three independent experiments performed in duplicate.

Conservation of the non-coding sequence, Up2, in five vertebrate species suggests functional conservation. To determine whether this enhancer element is active in mammalian hematopoietic cells, we performed transient transfection assays with a construct containing Up2. We transfected two human myeloid leukemia cell lines (KG-1 and K562) and a non-hematopoietic mammalian cell line NIH 3T3, with the mp/GFP, Up2-mp/GFP and the Up2a-mp/GFP constructs along with a CMV β-galactosidase plasmid to normalize for transfection efficiency. In myeloid cell lines, we observed increased GFP expression and this activity was stronger with the entire Up2 compared to activity of the minimal promoter (Figure 4D). Transfection with the Up2a element also showed increased GFP expression but this activity was less than that of the Up2. In both cases, the differences were found to be statistically significant when compared to the minimal promoter (Figure 4D). No GFP expression was observed in NIH3T3 cells; β-galactosidase expression was observed in this cell line indicating that Up2 and Up2a enhancer activities are specific to hematopoietic cells. Finally, we generated a reporter construct containing the mouse Up2 linked to GFP and analyzed its activity in transient transgenic zebrafish assays. As shown in Supplementary Table S (see additional file 2), mouse and zebrafish Up2 showed a similar enhancer activity to direct hematopoietic expression of GFP.

### Analysis of binding sites for potential transacting factors

Transfac6.0 Match and P-Match were used to search databases[20] for potential transcription factor binding sites involved in *GATA-2 *hematopoietic expression directed by Up2 sequence. Multiple transcription factor binding sites including those for AML1a, GATA-2, GATA-3, En-1, FAC1, HNF-1, E2F-1, LMO2, ARP-1 and HOXA3 were identified. Constructs carrying mutations on the binding sites for transcription factor AML-1, E2F-1, ARP-1, LMO2 and HOXA3 were generated and injected into zebrafish embryo for transient assays of hematopoietic GFP expression. Under a condition that is statistically significant, 19.49% (n = 318), 21.79% (n = 335) and 22.09% (n = 335) of the embryos injected with construct carrying mutation in E2F-1, LMO2 and HOXA3-A binding site, respectively, showed transient hematopoietic GFP expression (Figure 4A and 4B). These percentages are significantly lower than that of embryos injected with the control Up2 construct (35.2%). Other constructs did not show any significant differences (see additional file 2, Supplementary Table S).

EMSA showed that, *in vitro*, proteins of transcription factors E2F-1 and HOXA3 specifically formed the protein-DNA complexes with the probes containing LMO2 and HOXA3-A binding sites present in Up2, respectively (Figure 4C). With the anti-E2F-1 antibody, a super shift was observed while there was no super shift with a non-specific antibody. The shifted band was not observed with the probes containing a mutation in the E2F-1 and HOXA3-A binding site, respectively.

The function of E2F-1, LMO2 and HOXA3 binding sites were further analyzed in the germline transgenic zebrafish embryos carrying the described base change mutations in the same Up2-mp/GFP construct context. A base change mutation in E2F-1 binding site showed increased non-specific expression in muscle and notochord (Figure 3K) and less severe but notably reduced expression in hematopoietic cells. Mutation within LMO2 and HOXA3-A binding sites resulted in almost elimination of GFP expression in hematopoietic cells, showing a mosaic GFP positive pattern in less than 10% of hematopoietic cells compared to Up2-mp/GFP wild type construct (Figure 3L and see additional file 3, Supplementary Figure S2). As a control, mutation within the ARP-1 binding site showed no effect on GFP expression (Figure 3J). These results revealed that integrity of these putative LMO2 and HOXA3-A binding sites is required for proper regulation of *GATA-2/GFP *expression in hematopoietic cells.

## Discussion

Due to important roles of GATA-2 in hematopoiesis [1,8,9,21,22], a number of previous studies have been carried out to analyze the *cis*-acting elements and transcription factors that regulate hematopoietic *GATA-2 *expression. Transcription factors GATAs, Oct1 and Evi1 [4-6] and distal *cis*-acting DNA sequence elements appear involved in regulating its hematopoietic expression as revealed by transgenic animal studies [8,9]. The evolutionary conservation of non-coding sequences indicates that they have important biological functions, including developmental regulation of gene expression [23,24]. We used comparative genomics to identify three highly conserved non-coding sequences in the genomic regions flanking *GATA-2 *in the fugu, mouse, rat, human, and zebrafish genomes. One conserved element is located downstream, and two others, Up 1 and Up2, are located upstream of *GATA-2 *in a genomic region that, we showed in previous studies[9], is required for hematopoietic expression of *GATA-2 *in transgenic zebrafish. In this study we show that Up1 specifically directs GFP reporter gene expression in heart and Up2 is required for hematopoietic regulation of *GATA-2*. The enhancer activity of Up1 in heart should represent an authentic activity since *GATA-2 *is expressed in the mouse heart [8] as well as weakly expressed in embryonic zebrafish heart as detected by whole mount mRNA *in situ *hybridization (unpublished data).

Transgenic zebrafish carrying the Up2-mp/GFP construct expressed GFP strongly in hematopoietic tissues, showing that Up2 is sufficient to drive hematopoietic-specific gene expression. In addition, BAC clones having a deletion of Up2 did not drive hematopoietic gene expression whereas deletion of Up1 had no impact on hematopoietic gene expression, suggesting that Up2 is also necessary for regulating *GATA-2 *expression in hematopoietic tissues. In transfection analyses, we also found that hematopoietic Up2 enhancer activity is conserved in mammalian cells. Similarly, mouse Up2 enhancer has directed higher expression in zebrafish hematopoietic cells as shown by transient assays (Figure 4A, and see additional file 2, Supplementary table S), suggesting that function of Up2 is conserved between zebrafish and mammals. The tissue-specific expression in zebrafish appears to require elements that repress gene expression since deleting Up2b results non-specific expression of GFP in notochord (Figure 3H). Interestingly, we have also observed this in the regulation of the zebrafish *GATA-1 *gene [25].

Up2 contains several known consensus binding sites including those for AML1a, GATA-2, GATA-3, En-1, FAC1, HNF-1, E2F-1, LMO2 and HOXA3. Among these factors, HOXA3, AML1a, E2F-1, ARP-1, LMO2 and GATA-1/3 are likely to be involved in regulating hematopoietic *GATA-2 *expression since expression of the others has not been observed in hematopoietic tissues. Several HOX genes have recently been shown to be necessary for cdx4-mediated specification of hematopoietic cell fate during vertebrate embryogenesis[26]. Since specification of hematopoietic cell fate precedes hematopoietic *GATA-2 *expression, HOXA proteins are good candidates for *GATA-2 *regulation. E2F-1 and AML1a are also potential regulators of hematopoietic *GATA-2 *expression. *E2F-1 *mouse mutants have defects in hematopoietic progenitor cells that result from impeded cell cycle progression[27]. AML1, also known as Runx1, contributes to both transcriptional activation and repression in cell type-specific hematopoietic gene expression; *AML1 *knock-out mice lack fetal liver-derived hematopoietic cell clusters [28]. Furthermore, associated with chromosomal translocation, *AML1 *is involved in several types of acute myeloid leukemia[29]. ARP-1 is a regulator of gene switching from embryonic to fetal globins[30]. LMO2 forms transcription factor complexes with SCL and GATA factors in erythroid and hematopoietic progenitor cells[31]. GATA transcription factor binding sites found in Up2 may be involved in auto-regulation of *GATA-2 *expression by GATA-2[6] or *trans*-regulation by other GATA factors. GATA-1 and GATA-2 double knockdown in zebrafish did not eliminate initial expression of *GATA-2*[32] (also, personal communication from Zon LI, Children's Hospital, Boston). Similarly, *GATA-2 *promoter driven GFP expression were not affected in *GATA-2*^-/- ^or *GATA-3*^-/- ^null mouse embryos [6]. We therefore focused on analyzing function of binding sites for other non-GATA factors within up2. From the transgenic embryos injected with a series of site-specific mutagenesis constructs of other potential transcription binding sites contained in Up2, we demonstrated that the mutations in putative E2F-1, LMO2 and HOXA3-A binding sites decreased hematopoietic GFP expression. There are two high affinity HOXA3 binding sites in Up2 region. Mutation of the HOXA3-B binding site did not affect the hematopoietic expression of the GFP reporter gene, neither in transient nor in transgenic zebrafish embryos. Interestingly, this HOXA3-B site is not conserved from zebrafish to mammals. Thus, we consider the HOXA3-A binding site is the conserved functional site. Although mutations in the LMO2, E2F-1 and HOXA3-A binding sites all affect GFP expression in hematopoietic cells, LMO2 and HOXA3 sites appear playing a bigger role in positive regulation of *GATA-2 *whereas E2F1 site is more involved in restricting *GATA-2 *from expression in non-hematopoietic cells. Further analysis of their corresponding transcription factors by genetic and molecular approaches may lead to a better understanding of mechanisms underlying *GATA-2 *expression in hematopoietic cells.

Up2 is located near at 10 kb 5' of start codon in both the zebrafish and mouse *GATA-2 *gene. Previously, Kobayashi-Osaki et al. showed that a 26 base genomic sequence element located immediately 5' upstream of the conserved mouse up2 is essential for embryonic hematopoietic expression of *GATA-2*/GFP in mouse [6]. In addition, five of six GATA binding sites scattered in a genomic region of 336 bp, which encompass the up2 element, are critical for *GATA-2*/GFP expression in transgenic mouse. These observations, in addition to implicating roles of GATA factors in regulating hematopoietic *GATA-2 *expression, conceivably validate the necessary function of up2 in mouse. However, since expression of *GATA-2/GFP *is not affected in either *GATA-2*^-/- ^or *GATA-3*^-/- ^null mouse mutant as well as *GATA-2 *expression is not affected when both *GATA-1 *and *GATA-2 *are down regulated in zebrafish, other factors may play more essential roles in initiating embryonic *GATA-2 *hematopoietic expression. Our studies suggest that factors binding to putative HOXA3, LMO2 and E2F1 binding sites may be good candidates for such function. It is worth noting that the core sequence of HOXA3, LMO2 and GATA binding sites share high sequence similarity. Therefore the true identity of transcription factors binding to these sites *in vivo *remains as a subject of further investigation. Finally, mouse transgenic analysis suggests that a putative necessary enhancer for complete hematopoietic expression is located between 100–150 kb 5' of *GATA-2 *[8]. It would be interesting to determine in transgenic mouse if Up2 is also necessary by deletion analysis of BAC or YAC constructs. If yes, it would suggest that mouse needs additional enhancer located even further away to facilitate function of Up2 element in directing hematopoietic expression of *GATA-2*. *GATA-2 *regulation in mouse and zebrafish may certain differences reflecting evolutional divergence between the two species. Nonetheless, Up2 is conserved in sequence and genomic location and is functionally important in zebrafish.

Although we focus on analyzing regulation of *GATA-2 *gene expression the approach described here, drawing on embryological, genetic, molecular and computational methods, is generally applicable for rapidly identifying functional distal *cis*-acting sequences that regulate embryonic gene expression in vertebrates. In particular, we have established a transgenic line that expresses Tol2 transposase in germ cells, providing the transposase maternally eliminates the need to co-inject transposase mRNA. This simplified *Tol2 *transposon-mediated transgenesis approach allows generation of multiple stable transgenic zebrafish lines with non-mosaic expression of the GFP reporter gene. This study and a recent report by Fisher et al [33] showed that *Tol2 *transposon vectors are extremely useful for analyses of promoter and enhancer activity in zebrafish.

## Conclusion

A highly conserved 224 bp non-coding sequence (Up2) located approximately 10 Kb 5' upstream of the zebrafish *GATA-2 *is necessary and sufficient to direct hematopoietic expression of GFP reporter gene. Within Up2 element, binding sites for transacting factors HOXA3 and LMO2 appear important for its enhancer activity. Sequence similarity of Up2 among zebrafish, mouse and human genomes suggests function conservation.

## Methods

### Identification of highly conserved non-coding sequences in the genomic region bordering GATA-2

*Pipmaker *software was used to identify conserved non-coding sequences in genomic sequences flanking *GATA-2 *in mouse, rat, human and fugu and that of the zebrafish genome [34-36]. A 158 kbp zebrafish genomic sequence (Sanger Institute accession number: AL928619, Figure 1) was compared to orthologous regions of the other genomes. The genomic sequences from other species used for comparison were: rat chromosome 4 fragment 122,079,753 to 122,488,528 (~408 kbp); mouse chromosome 6 fragment 88,429,180 to 88,842,527 (~413 kbp); human chromosome 3 fragment 129,480,974 to 129,894,726 (~413 kbp) and fugu scaffold_374 (~189 kbp).

### Construction of Tol2 transposon vectors

Highly conserved zebrafish motifs (Up1, and Up2) identified by comparative genomics were amplified by PCR. The primers used in the PCR reactions were:

Up1 5' primer: 5'-ATCCGCTCGAG*TTTGACCTTTTCGGAAAACGAGCT*-3'

Up1 3' primer: 5'-CGGAATTC*TCTGATGGTCTCCACGGCAA*-3'

Up2 5' primer: 5'-ATCCGCTCGA*GATCTTCCCCCTCAAATGATGCA*-3'

Up2 3' primer: 5'-CGGAATTC*GGGGGTAATATTTCATCGGCGT*-3'

Up2a 3' primer: 5'-CGGAATTC*TGGAAACAGGGTCCAAAGT*-3'

Up2b 5' primer: 5'-ATCCGCTCG*AGCCTCGCTCGGTCCTGACA*-3'

The underlined bases correspond to restriction enzyme sites for Xho I (CTCGAG) or EcoR I (GAATTC); italicized bases are zebrafish genomic sequences. PCR products were digested with restriction enzymes Xho I and EcoR I and inserted proximal to a *GATA-2 *minimal promoter (mp)/GFP reporter gene contained in a *Tol2 *transposon vector (Figure 2G~L). The *GATA-2 *minimal promoter used is a 249 bp genomic sequence 5' proximal to the *GATA-2 *transcription start site. The *Tol2 *vector, kindly provided by Kochi Kawakami, was the original construct previously described [37].

Base change or deletion mutations were introduced into the putative binding sites of Up2 for transcription factors E2F-1, ARP-1, AML-1, LMO2 and HOXA3, respectively, using the mutagenesis kit from Strategene, Inc. (Catalog No. 200521). The primers used for the site-directed mutagenesis were:

AML-1 mF: 5'-ATTGAATGTATTTG*CGAGA*GCGGTTTGAAAAGGCGGCGA-3'

AML-1 mR: 5'-CCGCCTTTTCAAACCGC*TCTCG*CAAATACATTCAATGCA-3'

ARP-1 mF: 5'-CACCAATTTATTCAGCGCCAC*GGGA*GACCCTGTTTCCAGCCT-3'

ARP-1 mR: 5'-AGGCTGGAAACAGGGTC*TCCC*GTGGCGCTGAATAAATTGGT-3'

E2F-1 mF: 5'-CACCAATTTATTCAGC__ACTTTGGACCCTGTTTCC-3'

E2F-1 mR: 5'-GGAAACAGGGTCCAAAGT__GCTGAATAAATTGGTG-3'

LMO2 mF: 5'-CCAGCGGTAAGCT*ACGC*ACGATTAGGCCATTTATTATCT-3'

LMO2 mR:5'-GTGAAGATAATAAATGGCCTAATCGT*GCGT*AGCTTACCG-3'

HOXA3-A mF: 5'-TTTGAAAAGGCGGCGAT*GGC*CTGGGGGAGAGAGATAAG-3'

HOXA3-A mR: 5'-TTATCTCTCTCCCCCAG*GCC*ATCGCCGCCTTTTCAAACC-3'

HOXA3-B mF: 5'-CCAGCGGTAAGCTGATAACGA*CCC*GGCCATTTATTATCT-3'

HOXA3-B mR: 5'-GTGAAGATAATAAATGGCC*GGG*TCGTTATCAGCTTACC-3'

The underlined bases were the mutated sites. Three bases were deleted from E2F-1 binding site (shown as a line). Constructs carrying site-specific mutations were injected into wild type zebrafish embryos at one cell stage. The transient hematopoietic GFP expression was observed under fluorescent microscope after 24 hpf to calculate the percentage of hematopoietic GFP expression of each mutated construct.

### Construction of modified BAC constructs

BACs containing zebrafish *GATA-2 *identified in previous studies were used in the construction of modified BAC vectors[13,38]. Deletions of 5' upstream elements of *GATA-2 *were preformed using the shuttle vector pLD53.SCA_E_B as previously described [11,16]. In brief, 300~500 bp flanking sequences of Up1, or Up2, respectively, were amplified by PCR. The primers used in deletions of elements Up1 or Up2 or both were:

Up1 A box 5' primer: 5'-GCATGGCGCG*CCCCCCAAAAGTCACATCT*-3'

Up1 A box 3' primer: 5'-CCGGTTAATTAA*TTCCGAAAAGGTCAAACACA*-3'

Up1 B box 5' primer: 5'-CCGGTTAATTA*AGAGAGCGCGCGCCCGGTGAAGAT*-3'

Up1 B box 3' primer: 5'-CCGGATGGCCGGC*CTACTTAACAACTCCAACCCA*-3'

Up2 A box 5' primer: 5'-GCATGGCGCG*CCACTGGATAAAGGAGGCAA*-3'

Up2 A box 3' primer: 5'-ACGTTTAATTAA*GATCGCATGGGGTGATAGAGACAG*-3'

Up2 B box 5' primer: 5'-CCGGTTAATT*AAAGAGAGGGCCCTGGCATCT*-3'

Up2 B box 3' primer: 5'-CCGGATGGCCGGC*CGTTCAATGGACTCAAAAGGT*-3'

The underlined bases correspond to restriction enzyme sites for Asc I (GGCGCGCC), Pac I (TTAATTAA), or Fse I (GGCCGGCC), and italicized bases are zebrafish genomic sequences. PCR products were digested with restriction enzymes, and ligated into pLD53.SCA_E_B lacking the GFP reporter gene. The 5' flanking sequences were termed A boxes while the 3' flanking sequences as B boxes. After modification, the entire shuttle vector was integrated into the *GATA-2 *BAC clone by homologous recombination through one of the two homologous arms (A box or B box). Selection in LB media containing 5.5% sucrose resulted in deletions of Up1 or Up2 or both in approximately 50% of the BAC clones through recombination with the second homologous arm (B box or A box). Deletions were confirmed by sequencing.

The insertion of GFP into deletion and wild type *GATA-2 *BAC clones was carried out essentially as previously described [39]. In brief, a 377 bp PCR product, (A box) based on genomic sequence adjacent to and upstream of the zebrafish *GATA-2 *start codon, was inserted into the targeting vector pLD53.SC_2 _adjacent to a GFP reporter gene. The primers used in PCR amplification were:

5' primer: 5'-GATCGGCGCG*CCCGGTAGTTATTTGAAATTGCGA*-3'

3' primer: 5'-*CTCAAGTGTCCGCGCTTAGAAA*-3'

Underlined bases in the 5' primer correspond to restriction enzyme site for Asc I (GGCGCGCC) and italicized bases are zebrafish genomic sequences. In the presence of transiently expressed RecA, homologous recombination took place between the A box in the vector and the A box in the BAC clone resulting in integration of the vector insert into the BAC clone, placing the GFP reporter gene under control of the *GATA-2 *promoter within the clone (Figure 2B, D~F).

Modified *GATA-2 *BAC clones were identified by PCR using a forward primer (5'-CACGGGAAAAATAAACGCAGGA-3') upstream of the A box, and a reverse primer located in the GFP sequence (5'-GCTGCTTCATGTGGTCGGGGTA-3'). PCR positive clones were further confirmed by comparison of digestion patterns with that of the original BACs, or sequencing, or both.

### Gel mobility shift analysis

EST clone BC076469 (cDNA clone MGC: 91782, IMAGE: 7039846), encoding zebrafish HOXA3, was purchased from Open Biosystems, Inc. The ORF of HOXA3 was PCR amplified with primers:

5' primer: 5'-ATCGAGGATCCACATTGGGAAACGGCGAG AT-3'

3' primer: 5'-AACAAAATACACTGCGCCA-3'

The underlined bases in the 5' primer contained the BamH I restriction site. The PCR product was digested with BamH I and cloned into the vector GEX-2T digested with enzyme BamH I and Sma I to generate a HOXA3 in frame fusion protein with GST. Plasmid containing the GST-HOXA3 fusion protein was expressed in BL21(DE3)plyYs. The construct containing fusion protein of GST and human E2F-1 was a gift from Dr. Nevin's lab at Duke University[40]. Protein expression and purification were preformed as previously described[41].

Electrophoretic Mobility Shift Analysis (EMSA) was preformed to confirm the binding affinity of the transcriptional factors with Up2 sequence with non-radioactive gel shift kit (Roche Applied Sciences, Inc. Catalog number: 03353591910). Complementary strand DNA oligoes containing transcription factor E2F-1 and HOXA3-A binding site, respectively, were annealed and DIG labeled. The sequences are as follow:

HOXA3-A probe: 5'-GGTTTGAAAAGGCGGCGATAATCTGGGGGAGAGAGATAA-3'

E2F1 probe: 5'-ACACCAATTTATTCAGCGCCACTTTGGACCCTGTTTCCA-3'

Mouse monoclonal antibody KH95 (Abcom, Inc) was used for super shift analysis and chicken anti-red fluorescent protein (RFP) polyclonal antibody (Chemicon International, Inc. Catalog No. AB3528) was used as non-specific antibody.

### Generation of transgenic zebrafish

*Tol2 *vector-based plasmids were extracted and purified using a Qiaprep Spin Miniprep Kit (Qiagen). Modified BAC clone DNA was extracted using Hi-speed Maxi Prep Kit (Qiagen). BAC DNA was digested with restriction enzyme Not I and dialyzed in 0.5 × TE [13,14]. DNA concentrations were adjusted to ~100 ng/ul in 0.1 M KCl.

BAC construct DNA was microinjected into wild type zebrafish fertilized eggs as previously described [38], resulting 4–7% germline transmission. *Tol2 *constructs were injected into transgenic zebrafish fertilized eggs containing maternally expressed Tol2 transposase [37] driven by the zebrafish *vasa *promoter [42]. F1 generation transgenic zebrafish embryos were identified by PCR. Fluorescent expression patterns were observed using a compound fluorescent microscope (Axioplan Imaging, Zeiss). Photographs were captured using an AxioCam digital camera (Zeiss), and Openlab software (Improvision).

### Analysis of GFP reporter gene expression in mammalian cells lines

Transient transfection assays were performed in human myeloid leukemia cell lines, KG1 and K562, and in mouse fibroblasts NIH3T3. KG1 and K562 cells were cultured in Iscoves media containing 10% fetal bovine serum (FBS), 2 mM/L L-glutamine, 100 U/ml Penicillin and 100 mg/mL Streptomycin. NIH3T3 and 293T cells were cultured in DMEM supplemented with 10% FBS, 2 mM/L L-glutamine, 100 U/ml Penicillin and 100 mg/mL Streptomycin. Fifteen micrograms of *GATA-2*/GFP fusion constructs were co-transfected with 5 ug of the CMV-β-galactosidase plasmids into mammalian cells by electroporation as previously described[43,44]. Forty-eight hours after transfection, cells were harvested and β-galactosidase activity was measured using the Promega assay kit (Promega). GFP expression levels were determined using flow cytometry. 10^6 ^Cells were harvested, washed, and resuspended in phosphate-buffered saline (PBS) and analyzed using a FACS-SCAN cytometer (Beckton-Dickinson). The percentage of GFP positive cells was calculated using cell quest software and expression levels were normalized using CMV-β-galactosidase activity. P values were calculated using the Student-T test method and Jump In software.

## Authors' contributions

ZY conducted most of the experiments. HJ participated in BAC modification and generation of transposon plasmids and transgenic zebrafish. FZ and MZ carried out the bioinformatics analysis. DS and KS conducted experiment of transfection assays and statistic analysis. SL conceived the study, and participated in its design and coordination. ZY and SL wrote the manuscript. All authors read and approved the final manuscript.

## Supplementary Material

Additional file 1 5' RACE of zebrafish *GATA-2 *mRNA. The 5'RACE result shown single RT-PCR product. Click here for file

Additional file 2 Supplementary Table S: GFP Hematopoietic Transient Expression. Results of GFP hematopoietic transient expression from zebrafish embryos that injected with DNA constructs containing wild type or base change mutated Up2 at one cell stage. Click here for file

Additional file 3 Mutation in LMO2 Binding Site Reduced GFP Hematopoietic Expression. Transgenic zebrafish embryos carrying base change mutation in the binding site of LMO2 significantly reduced GFP hematopoietic expression. Click here for file
