# Supplementary material for: A highly conserved regulatory element controls hematopoietic expression of GATA-2 in zebrafish
Source: BMC Dev Biol. 2007 Aug 20;7:97. doi: 10.1186/1471-213X-7-97 (PMC1988811; doi:10.1186/1471-213X-7-97)
Supplement: Additional file 1 — 5' RACE of zebrafish GATA-2 mRNA. The 5'RACE result shown single RT-PCR product. [file 1471-213X-7-97-S1.pdf]

Additional file1

5' RACE of Zebrafish *GATA-2* mRNA. To determine if zebrafish *GATA-2* locus, uses alternative first exon 5' RACE was preformed using FirstChoice RLM-RACE Kit (Ambion, Inc., Cat# 1700) and combined with *GATA-2* gene specific 3' reversed primers which are located in the 2nd exon of zebrafish *GATA-2* and downstream of the start codon. The sequences of the 3' reversed primers are:
Outer 3' reversed primer: 5'-ACAATTACATGGAGCCCATGGC-3';
Inner 3'reversed primer: 5'-ATCAGTCTCGATGGATGGCCCA-3'

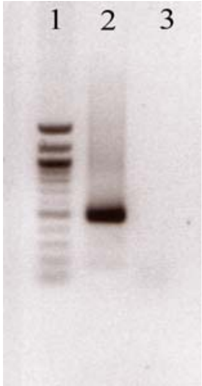

Figure S1. Single RT-PCR product was observed after the nested PCR with commercial 5'RACE primer and zebrafish *GATA-2* inner 3' reversed primer. Sequencing results shown that the RT-PCT product is as same as that previously reported. 1, 100bp ladder from New England Biolabs, Inc. 2, RT-PCR product after the 2nd round nested PCR. 3, Negative control for RT-PCR.
