# Supplementary material for: A highly conserved regulatory element controls hematopoietic expression of GATA-2 in zebrafish
Source: BMC Dev Biol. 2007 Aug 20;7:97. doi: 10.1186/1471-213X-7-97 (PMC1988811; doi:10.1186/1471-213X-7-97)
Supplement: Additional file 2 — Supplementary Table S: GFP Hematopoietic Transient Expression. Results of GFP hematopoietic transient expression from zebrafish embryos that injected with DNA constructs containing wild type or base change mutated Up2 at one cell stage. [file 1471-213X-7-97-S2.doc]

**Additional file2**

**Supplementary Table S: GFP Hematopoietic Transient Expression.**

|  | zUp2^*^ | mUp2^**^ | zUp2 mutations | | | | | | |
| --- | --- | --- | --- | --- | --- | --- | --- | --- | --- |
|  |  |  | HoxA3-A | HoxA3-B | LMO2 | E2F-1 | ARP-1 | ALM-1 | E2F-1/HoxA3-A |
| n | 267 | 379 | 335 | 213 | 335 | 318 | 271 | 220 | 345 |
| Positive | 94 | 130 | 74 | 66 | 73 | 62 | 92 | 85 | 52 |
| Percentage | 35.20 | 34.30 | 22.09 | 31.00 | 21.79 | 19.49 | 33.94 | 38.63 | 15.07 |

*: Conserved Up2 domain from zebrafish *GATA-2* locus linked with zebrafish *GATA-2* minimal promoter.

**: Conserved Up2 domain from mouse *GATA-2* locus linked with zebrafish *GATA-2* minimal promoter.
