# Supplementary material for: A highly conserved regulatory element controls hematopoietic expression of GATA-2 in zebrafish
Source: BMC Dev Biol. 2007 Aug 20;7:97. doi: 10.1186/1471-213X-7-97 (PMC1988811; doi:10.1186/1471-213X-7-97)
Supplement: Additional file 3 — Mutation in LMO2 Binding Site Reduced GFP Hematopoietic Expression. Transgenic zebrafish embryos carrying base change mutation in the binding site of LMO2 significantly reduced GFP hematopoietic expression. [file 1471-213X-7-97-S3.pdf]

Additional file3

Mutation in LMO2 Binding Site Reduced GFP Hematopoietic Expression.

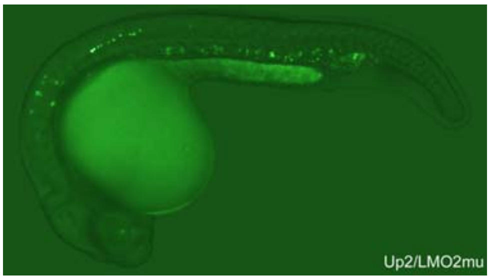

Figure S2. Transgenic zebrafish embryos carrying base change mutation in the binding site of LMO2 significantly reduced GFP hematopoietic expression.
